# Wide spetcrum mutational analysis of metastatic renal cell cancer: a retrospective next generation sequencing approach

**DOI:** 10.18632/oncotarget.12551

**Published:** 2016-10-10

**Authors:** Michelangelo Fiorentino, Elisa Gruppioni, Francesco Massari, Francesca Giunchi, Annalisa Altimari, Chiara Ciccarese, Davide Bimbatti, Aldo Scarpa, Roberto Iacovelli, Camillo Porta, Sarhadi Virinder, Giampaolo Tortora, Walter Artibani, Riccardo Schiavina, Andrea Ardizzoni, Matteo Brunelli, Sakari Knuutila, Guido Martignoni

**Affiliations:** ^1^ Laboratory of Oncologic Molecular Pathology, S.Orsola-Malpighi Hospital, Bologna, Italy; ^2^ Department of Medical Oncology, S.Orsola-Malpighi Hospital, Bologna, Italy; ^3^ Department of Urology, S.Orsola-Malpighi Hospital, Bologna, Italy; ^4^ Department of Pathology, University of Helsinki, Faculty of Medicine, Helsinki, Finland; ^5^ Department of Pathology, Diagnostics and Public Health University of Verona, Verona, Italy; ^6^ Department of Urology, Diagnostics and Public Health University of Verona, Verona, Italy; ^7^ Department of Medical Oncology, Diagnostics and Public Health University of Verona, Verona, Italy; ^8^ Diagnostics and Public Health University of Verona, Verona, Italy; ^9^ Department of Medical Oncology, University of Pavia, Pavia, Italy

**Keywords:** renal cell carcinoma, next generation sequencing, target therapy, metastatic disease, VHL, Pathology Section

## Abstract

Renal cell cancer (RCC) is characterized by histological and molecular heterogeneity that may account for variable response to targeted therapies. We evaluated retrospectively with a next generation sequencing (NGS) approach using a pre-designed cancer panel the mutation burden of 32 lesions from 22 metastatic RCC patients treated with at least one tyrosine kinase or mTOR inhibitor. We identified mutations in the *VHL, PTEN, JAK3, MET, ERBB4, APC, CDKN2A, FGFR3, EGFR, RB1, TP53* genes. Somatic alterations were correlated with response to therapy. Most mutations hit *VHL1* (31,8%) followed by *PTEN* (13,6%), *JAK3, FGFR* and *TP53* (9% each). Eight (36%) patients were wild-type at least for the genes included in the panel.

A genotype concordance between primary RCC and its secondary lesion was found in 3/6 cases. Patients were treated with Sorafenib, Sunitinib and Temsirolimus with partial responses in 4 (18,2%) and disease stabilization in 7 (31,8%). Among the 4 partial responders, 1 (25%) was wild-type and 3 (75%) harbored different *VHL1* variants. Among the 7 patients with disease stabilization 2 (29%) were wild-type, 2 (29%) *PTEN* mutated, and single patients (14% each) displayed mutations in VHL1, *JAK3* and *APC/CDKN2A*. Among the 11 non-responders 7 (64%) were wild-type, 2 (18%) were p53 mutated and 2 (18%) *VHL1* mutated.

No significant associations were found among RCC histotype, mutation variants and response to therapies. In the absence of predictive biomarkers for metastatic RCC treatment, a NGS approach may address single patients to basket clinical trials according to actionable molecular specific alterations.

## INTRODUCTION

Renal cell carcinoma (RCC) accounts for the 2-3% of all adult malignancies, the seventh most common cancer in men and the ninth in women. [1] Localized RCC can be successfully cured by surgery in most cases. Unfortunately, about 30% of patients relapse after nephrectomy or present at diagnosis with metastatic disease (mRCC)[2]. Despite the therapeutic improvements made in the past decade, mRCC is still an incurable disease. [3]

Several studies outlined that the multiple histological features of RCC corresponded to a molecular intra- and inter-tumoral heterogeneity with prognostic and predictive implications.[3, 4, 5, 6, 7] The improved knowledge of the biology and pathogenesis of RCC, lead to major treatment advances through the development of targeted agents. [2] The currently available therapeutic options for mRCC include drugs targeting circulating vascular endothelial growth factor (VEGF) and its receptors (VEGFRs), other tyrosine kinase inhibitors (TKIs) and inhibitors of the mammalian target of rapamycin (mTOR) serine-threonine kinase [2]. Unfortunately, 20 to 30% of mRCC patients do not respond at all to targeted agents while the remaining ultimately progress after an initial benefit. [8] There is current need for molecular biomarkers of RCC for the development of a personalized treatment and predictive approach for patient stratification. [8] According to the Cancer Genome Atlas database the most frequent somatic mutations in ccRCC include mainly alterations of the *VHL* gene and its partners involved in neo-angiogenesis and response to hypoxia, followed by alterations of the PI(3)K/AKT/mTOR pathway. [9] Conversely, the most frequent somatic genetic changes in non-ccRCC such as the papillary histotype (pRCC) involve mutations or copy number variations of the *MET* oncogene or loss of the tumor suppressor gene *CDKN2A.* [3, 10]

Unfortunately, no correlation was found so far among biomarker expression/alteration and clinical response to single TKIs and mTOR inhibitors. In addition, there is no clear-cut evidence that the ccRCC or the non-ccRCC histotypes respond differently to specific target therapies [9, 11]

In this study, we have evaluated retrospectively with a next generation sequencing (NGS) approach the mutation burden of 32 primary and/or secondary lesions of 22 patients with mRCC treated with at least one TKI or mTOR inhibitor. We then associated the presence of every identified somatic tumor alteration with response to therapy.

### Patients’ selection criteria

We have retrospectively analyzed 32 RCC nodules/metastases from 22 patients with mRCC enrolled and surgically resected at the S.Orsola-Malpighi Hospital (Bologna) and the Policlinico GB Rossi Hospital (Verona) from 2001 to 2013. Informed consent for genetic analyses was obtained from the patients before surgery.

Selection criteria for patients’ enrolment were: 1) presence of mRCC at the time of surgery or development of metastases thereafter; 2) Availability of at least one formalin-fixed paraffin-embedded (FFPE) surgical pathology cancer specimen; 3) Systemic treatment with at least one of the anticancer agents registered in Italy for mRCC; 4) availability of at least 6 month follow-up from the beginning of the first line of treatment. When available, tissues from the primary and metastatic tumor sites were analyzed separately. In addition, histological areas of sarcomatoid or rhabdoid change within the same tumor nodule were also analyzed individually. Table 1 summarizes the clinical-pathological features of the selected cases.

**Figure 1:**
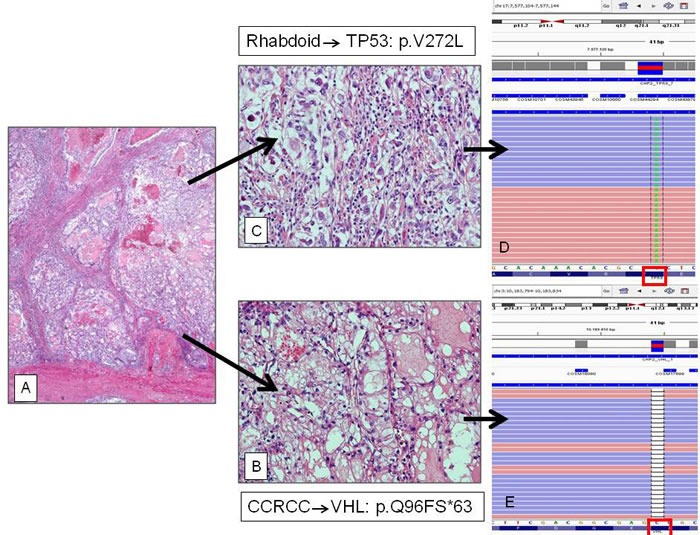
A) Low power (2X) of a RCC with B)clear–cell and C) rhabdoid components (20X) and two different mutational patterns D) The rhabdoid component showed the p.V272L hot-spot mutation of the *TP53* gene; while E) the clear cell counterpart harbored the missense p.Q96FS*63 mutation of the *VHL1* gene.

**Table 1:** Clinical-pathological patients’characteristics according to mutation variants and line of therapy. PR: partial response; PD: progression disease; SD: stable disease

PATIENT	AGE	SEX	TISSUE	HISTOTYPE	TIME to metastasis	I-line THERAPY	RESPONSE after I-line therapy	II-line THERAPY	RESPONSE after II-line therapy	III-line THERAPY	Long survivors	GENE/MUTATION/VARIANTS
1	74	M	kidney	ccRCC	0	Sorafenib	PR	Sorafenib	-	-	-	FGFR3	VHL1	PTEN
			diaphragm									FGFR3	VHL1	PTEN
2	56	F	kidney		27	Sunitinib	PR	Everolimus	-	-	-	VHL1	MET	
			lung	ccRCC								VHL1	MET	
3	59	M	adrenal gland		0	Sorafenib	PD	Sorafenib	-	-	-			
			kidney	ccRCC									
4	48	F	liver	ccRCC	0	Sorafenib	PD	Sorafenib	-	-	-			
5	66	F	kidney	ccRCC	1	Sunitinib	PD	-	-	-	-				6	64	M	kidney	ccRCC	41	Sorafenib	PR	Everolimus	-	-	-
			pancreas																							
7	58	M	kidney	ccRCC	37	Sunitinib	SD	Everolimus	-	-	-
			JAK3										lung		JAK3		
8	50	M	kidney	ccRCC	53	Sunitinib	PD	-		-	+ 59mo	ERBB4		
9	46	M	kidney	ccRCC	0	Sunitinib	PD	Sarafenib	PD	-	-			
10	35	M	lymph-node	pRCC	13	Sunitinib	PD	-		-	-			
11	51	M	kidney	pRCC	11	Sunitinib	SD	Everolimus	PD	Sorafenib	-	APC	CDKN2A	
12	74	M	kidney	ccRCC	9	Sunitinib	SD	Everolimus	PD	-	-			
13	46	M	kidney (rhadboid component)	ccRCC	14	Sunitinib	SD	Everolimus	PD	Sorafenib	LFU 30 mo	EGFR	RB1	
			liver	PTEN									
14	66	M	omentum	ccRCC	32	Sorafenib	SD	-		-	-			
15	55	M	kidney (rhadboid component)	ccRCC	0	Temsirolimus	PD	Sunitinib		-	-	TP53		
			kidney	VHL1									
16	68	M	kidney	ccRCC	9	Sunitinib	SD	-		-	+ 27 mo			
			kidney	pRCC								PTEN		
17	62	M	kidney (rhadboid component)	ccRCC	0	Sunitinib	PD	Everolimus	PD	Sorafenib	-	FGFR3	VHL1	
			kidney	FGFR3								VHL1	
18	37	F	kidney (sarcomatoid component)	ccRCC	0	Sunitinib	PD	Everolimus	PD	Sorafenib	-	VHL1	JAK3	
			kidney (rhadboidcomponent)									VHL1	JAK3	
19	79	F	kidney	ccRCC	14	Sunitinib	SD	Axitinib	SD	Sorafenib	-	VHL1		
20	34	M	kidney	ccRCC	0	Sunitinib	PR	-		-	+ 6mo	VHL 1		
21	70	M	lymph node	ccRCC	36	Sorafenib	PD	Everolimus		-	+ 60mo			
22	62	M	liver	pRCC	0	Sunitinib	PD	Everolimus	PD	Sorafenib	-	TP53		

**Table 2:** Mutation variants according to gene, tumor site, and rhabdoid or sarcomatoid component

Genes	Variation	Frequency	# of patients	metastasis	kidney	sarcomatoid component	rabdhoid component	histology	Response to therapy
VHL	p.S80N (c.239G>A)	10%	1	1	1			ccRCC	PR
	p.P86T (c.256C>A)	16,3%	1	1	1			ccRCC	PR
	p.Q96fs*63 (c.286delC)	23,0%	1		1			ccRCC	PD
	p.P97R (c.290delCCinsGT)	41,9%-52,6%	1			1	1	ccRCC	PD
	p.V137fs*7 (c.405_406insT)	41,6%-32,1%	1		1		1	ccRCC	PD
	p.F91fs*68 (c.271delT)	40,0%	1		1			ccRCC	SD
	p.W117fs*15 (c.346_347insT)	49,1%	1		1			ccRCC	PR
PTEN	1089624295T>TA	11,9%	1	1	1			ccRCC	PR
	p.L320S (c.959T>C)	43,5%-72,8%	2	1	1			ccRCC/pRCC	SD
JAK 3	p.V722I (c.2164G>A)	49,1%-47,3%-51,6%	2	1	1	1	1	ccRCC	SD/PD
MET	p.R988C (c.2962C>T)	49,4%	1	1	1			ccRCC	PR
ERBB4	p.S303Y (c.908C>A)	35,2%	1		1			ccRCC	PD
APC	p.P1433L (c.4298C>T)	100%	1		1			pRCC	PD
CDKN2A	p.H83Y (c.247C>T)	10%	1		1			pRCC	PD
FGFR3	p.F386L (c.1156T>C)	38,8%-26,9%-48,3%	2	1	2		1	ccRCC	PR/PD
EGFR	p.G873R (c.2617G>A)	12,40%	1				1	ccRCC	SD
RB1	p.I680T (c.2039T>C)	10,50%	1				1	ccRCC	SD
TP53	p.V272L (c.814G>T)	17%	1				1	ccRCC	PD
	p.T172I (c.632C>T)	36,40%	1	1				ccRCC	PD

ccRCC: clear cell renal cell carcinoma; pRCC: papillary renal cell carcinoma; PR: partial response; PD: progression disease; SD: stable disease

### Samples’ preparation and NGS sequencing

Pathological slides from all patients and all tumor sites have been reviewed blindly by four genito-urinary dedicated pathologists (MB, MF, FG and GM) for independent assessment of RCC histotype, grading and staging, according to the 2016 WHO classification of tumours of the urinary system and male genital organs [12]. Histological diagnosis was confirmed by routine immunohistochemistry in all cases using antibodies anti CA-IX and RCC for ccRCC and anti CK7 and Racemase for pRCC. Histotype call was performed blindly by at least two pathologists with inter-observer variability <5%. (data not shown)All the slides of each case have been pathologically reviewed and the most representative block with highest tumor cell enrichment and highest nucleolar grade was selected. Tumor areas of interest with at least 70% tumor cell enrichment were circled and 10 µm-thick serial sections of the same paraffin block cut in sterility for DNA extraction. Sections were manually microdissected, deparaffinized in xylene and the DNA was isolated using the GeneRead DNA FFPE Kit (Qiagen - Hilden, Germany). DNA was quantified with the Quantifiler^®^ Human DNA Quantification Kit (Thermo Fisher Scientific -Waltham, MA). The NGS analysis was performed on an Ion PGM™ System (Thermo Fisher Scientific) platform. An amplicon library was produced from 10ng of DNA from each sample using the Ion AmpliSeq™Cancer Hotspot Panel v2 that generates 207 amplicons encompassing hotspot and targeted regions of 50 genes *(ABL1, EGFR, GNAS, KRAS, PTPN11, AKT1, ERBB2, GNAQ, MET, RB1, ALK, ERBB4, HNF1A, MLH1, RET, APC, EZH2, HRAS, MPL, SMAD4, ATM, FBXW7, IDH1, NOTCH1, SMARCB1, BRAF, FGFR1, JAK2, NPM1, SMO, CDH1, FGFR2, JAK3, NRAS, SRC, CDKN2A, FGFR3,IDH2,PDGFRA,STK11,CSF1R,FLT3,KDR,PIK3CA,TP53,CTNNB1,GNA11,KIT,PTEN,VHL)*. Amplification of target sequences was followed by barcode adapter ligation (Ion Xpress Barcode Adapters) to the amplicons that were then purified using Agencourt AMPure XP (Beckman Coulter - Brea, California). Library quantification was performed using the Ion Library TaqMan™ Quantitation Kit. The library was diluted in nuclease-free water to obtain a final concentration of 8pM. Emulsion PCR was performed using Ion PGM™ Template OT2 200 Kit on the Ion OneTouch™ 2 instrument. Library quality was assessed using the Qubit Ion Sphere™ Quality Control Kit. Selective ion spheres (ISPs) with DNA were isolated (Ion PGM™ Enrichment Beads on Ion OneTouch™ ES instrument) and sequenced on a Ion 316™ Chip Kit v2 (5 samples/chip) or a Ion 318™ Chip Kit v2 (10 samples/chip) using the Ion PGM™ Sequencing 200 Kit v2. Successful sequencing of a sample required at least 500 000 reads with a quality score ≥ Q20. As tumor specimens were admixed with normal tissue, a minimum coverage of 500X with at least 10% frequency was used as cut-off for a variant to be considered true.

### NGS data analysis

Sequence alignment and base calling were performed using the Torrent Suite software v.4.4.3 (Thermo Fisher Scientific) taking Human Genome Build 19 (hg19) as the reference. Variant calling was carried out with the Variant Caller v.4.4.3.3 plug-in using default “Somatic—Low Stringency” settings. Variants were further filtered using Ion Reporter software v.4.4 (Thermo Fisher Scientific).

The following stringent criteria were applied for final variant calling: i) non-synonymous coding; ii) allele frequency of ≥10%; iii) total amplicon coverage of ≥500 reads; iv) a Phred-based quality score of 30 or more and P-value <0.01. The Ion Reporter software incorporated the following databases: ClinVar, dpSNP (National Center for Biotechnology Information, Bethesda, Maryland) and COSMIC (Wellcome Trust Sanger Institute, Cambridge, United Kingdom) databases. The in-silico prediction tools SIFT, PolyPhen, PhyloP and Grantham were also included. The Integrative Genomics Viewer (IGV, Broad Institute) was used to visualize variants.

## RESULTS

### Clinical-pathological data

Seventeen (85%) of the 22 mRCC patients were males and five (15%) females. The mean age at diagnosis was 57±19. Twenty-one of the 31 RCC samples were from the primary tumor site and 11 from metastatic lesions. The mean time to the development of metastases from the removal of the primary tumor was 13.5 ±14.27 months (range from 0, synchronous, to 53 months). RCC histotypes were distributed as follows: 19 (86%) ccRCC and 4 (18%) papillary RCC (pRCC).. One patient developed two nodules with ccRCC and pRCC histology in the same kidney and the lesions were analyzed separately. Metastatic sites included lymph-nodes, diaphragm, liver, pancreas, omentum, lung and adrenal. Both the primary and the metastatic sites were analyzed for 6 patients. The rhabdoid and the sarcomatoid tumor components were present and have been tested in 4 and in 1 sample respectively (Table 1).

### Mutation variants are different, non-mutually exclusive, and variably associated with pathological features

The mean read length of the NGS was 105 bp and the average reads were approximately 65Mb of sequence per sample. With normalization to 2000 reads per specimen, 97.08% amplicons averaged at least 500 reads. A minimum coverage of 500X with at least 10% frequency was used as cutoff for a variant to be considered true. We identified mutations in the genes *VHL, PTEN, JAK3, MET, ERBB4, APC, CDKN2A, FGFR3, EGFR, RB1* and *TP53*.

Table 1 summarizes the clinical-pathological patients’ characteristics according to mutation variants and the line of therapy. In particular, the most frequent mutations (7/22 patients 31,8%) affected the *VHL1* gene, all were in different loci and except for 3 cases were always associated with other mutations. *VHL1* was mutated in 6 primary RCC, in 2 metastatic sites and in 1 sarcomatoid and 2 rhabdoid components of primary RCCs. The *VHL1* mutation variant was concordant between primary and the corresponding metastasis in 1 out of 2 cases. *PTEN* was found mutated in 3/22 (13,6%) patients and only in 1 case was associated with mutations of *VHL 1* and *FGFR3*. Two cases harbored the same *PTEN* variant (L320S). Two patients (2/22) ( 9%) showed the same *JAK3* mutation variant (V722I); it was associated with a *VHL 1* mutation and was revealed both in the sarcomatoid and rhadboid component of the same patient, while in the other patient it was present as a single mutation in the primary RCC and the metastatic lesion. Two patients (9%) harboured a mutation of the *FGFR3* gene (F386L); in both cases it was associated with a *VHL1* mutation (1 cases also with a *PTEN* mutation) and each patient presented respectively the mutation both in the primary RCC and the corresponding metastasis and the clear cell and in the rhabdoid component. *TP53* was found mutated in 2/22 (9%) patients:1 in a metastatic lesion and 1 in the rhabdoid component of a primary RCC. Mutation in *MET* gene was found in 1/22 patients (4,5%) in the primary RCC and in association with *VHL1* mutation. *ERBB4* mutation were discovered in 1/22 (4,5%) patients in both primary and metastatic RCC sites, in association with *VHL1* mutation. Double mutations in the *APC* and *DCKN2A* genes was found in the same patient in the primary RCC. Mutations of *EGFR* and *RB1*genes were both detected in 1 patients in the rhabdoid component of the primary RCC.

In 9/22 (20%) patients the tumors were wild type at least for the investigated genes. There was no relevant association between RCC histotype and mutation variants. In particular the four pRCC harbored mutations in the *APC, DCKN2A, PTEN* and *TP53* genes but not in *VHL1*. Eight (42%) out of the remaining 19 ccRCC tumors habored at least one *VHL1* mutation although with different variants.

As depicted in Table 3, the concordance between the primary RCC and its rhadboid/sarcomatoid component was seen in 2/3 cases: *FGFR3* F386L and *VHL1* V13fs*7 (rhabdoid and primary RCC) and *VHL1* P97R and *JAK3* (rhabdoid and sarcomatoid component of RCC). In the remaining case the gene mutations between the RCC and its rhabdoid component were completely different. The concordance between RCC and its metastatic lesion was found in 3/6 cases while in the remaining case the genetic alterations were different.

**Table 3:** Concordance of mutation variants according to primary/secondary tumor site or histological component

PATIENT	Tumor site/component	GENE/MUTATION/VARIANTS		
1	kidney	FGFR3	VHL1	PTEN
	diaphragm	FGFR3	VHL1	PTEN
2	kidney	VHL1	MET	
	lung	VHL1	MET	
3	adrenal gland			
	kidney			
6	kidney			
	pancreas			
7	kidney	JAK3		
	lung	JAK3		
13	kidney (rhadboid)	EGFR	RB1	
	liver	PTEN		
15	kidney (rhadboid)	TP53		
	kidney	VHL1		
16	kidney			
	kidney	PTEN		
17	kidney (rhadboid)	FGFR3	VHL1	
	kidney	FGFR3	VHL1	
18	kidney (sarcomatoid)	VHL1	JAK3	
	kidney (rhadboid)	VHL1	JAK3	

### Response to target therapy is irrespective of mutation variants

Table 1 depicts response to therapy for each patient while Table 2 summarizes the mutation variants found by gene, tumor site, and rhabdoid or sarcomatoid component.

Six (27%) mRCC patients underwent first-line therapy with Sorafenib, 15 (68%) with Sunitinib and 1 (4%) with Temsirolimus. Radiological partial response (PR) was obtained in 4 (18,2%) patients after at least one cycle of first-line therapy with TKI (2 after Sunitinib and 2 Sorafenib). Disease stabilization (SD) as best response was reached in 7 (31,8%) patients (6 after Sunitinib and 1 after Sorafenib), while the remaining 11 (50%) patients experienced early disease progression (7 after Sunitinib, 3 after Sorafenib and 1 after Temsirolimus).

Among the 4 partial responders, 1 (25%) was wild-type for all genes and 3 (75%) harbored different *VHL1* variants. Among the 7 patients with disease stabilization 2 (29%) were wild-type for all genes, 2 (29%)were *PTEN* mutated, and mutations in single patients (14% each) were found for *VHL1, JAK3* and *APC/CDKN2A*. Twelve (55%) patients underwent second-line therapy after progression to first-line agents: 4 (18%) with Sorafenib, 1 (4%) with Sunitinib and 7 (32%) with Everolimus. None of these patients showed partial response after second-line therapy with just one case of disease stabilization after Axitinib.

Among the 11 non-responders 7 (64%) were wild-type for all genes, 2 (18%) were *TP53* mutated (in different loci) and 2 (18%) were *VHL1* mutated.

The mean follow-up time was 27,5 months (range 6-78). Four of the 22 (18,2%) patients were alive at the time of last follow-up and only 1 was lost from follow up after 30 months. Of these 5 long survivors 4 (80%) were treated with Sunitinib and 1 (20%) with Sorafenib. Two patients harbouring just the same *PTEN* L320S mutation survived for at least 27 or 30 months.

## DISCUSSION

Renal cell cancer is as an heterogenous disease based on its morphological and molecular features. The ccRCC is largely the most common histotype and it is tightly associated with alteration of the *VHL1-HIF* pathway, while non-clear cell tumors are characterized by alterations of the *PI3K* and *MET* pathways. However, a large morphological and molecular inter-tumor heterogeneity may occur within the same histotype and intra-tumor in primary and metastatic lesions of the same patient. [4]

Here we provided one of the few available reports on wide spectrum mutation analysis on mRCC on an NGS platform. Although we did not find a significant association between histotype and mutation variants we found that approximately 40% of ccRCC harboured mutations of *VHL1* gene compared to none in the pRCC group. We found a large number of mutations in different genes. Mutations in some genes such as *VHL1*, *PTEN, JAK3* and *TP53* are well recognized. [13, 14, 15] By contrast, mutations in the genes *APC, ERBB4, RB1, EGFR, FGFR3* have been rarely or never found in RCC but were encountered in our series. [16] As far as we can say from our data these mutations are non-mutually exclusive and hit a variable number of loci. In addition, we did not find any significant correlation between sarcomatoid or rhabdoid RCC features and specific gene alterations while concordance between primary RCC and the corresponding metastasis was found in three of six cases. Taken together all these data bring to the conclusion that more gene variants than expected are associated with mRCC, and are non-mutually exclusive. Mutations of *VHL1* are the most frequent but involve wide number of loci.

Major limitations affect the present study. The limited number of cases and the even smaller number of patients with available primary tumors and metastases do not allow drawing definitive conclusions on the intra-tumor heterogeneity in terms of both mixed histotypes or primary/metastases variability. In addition, our mutational analysis is based on a panel generating 207 amplicons covering approximately 2,800 COSMIC mutations from 50 of the most altered oncogenes and tumor suppressor genes in solid tumors. We may have missed mutations in the genes outside the list of our panel as well as genetic rearrangements detectable just at the RNA level. Larger studies covering all the RCC histotypes with paired samples of primary tumors and metastases are required to answer the intra-tumor heterogeneity question.

Since 70 to 80% of patients obtain a benefit from a first line therapy, a large (and presently increasing) number of mRCC patients are treated sequentially with different agents. [17] Unfortunately, there are no current validated predictive biomarkers of response to these target therapies. Our retrospective analysis confirmed that there are no single predictors of response to therapy with TKIs or mTOR inhibitors. In fact, we found that three of the four patients experiencing partial response after first-line therapy were treated with a TKI with anti-angiogenetic activity and actually harbored mutations of the pro-angiogenic gene *VHL1*, although in different loci. By contrast, other three patients with tumors harboring *VHL1* mutations experienced progressive disease with similar therapies. Although in few patients, this finding is in line with other studies reporting no association between *VHL1* mutations and objective responses to anti-angiogenic TKIs. [18, 19] Interestingly, seven (64%) of the 11 mRCC patients who did not respond even at the first-line treatment were completely wild-type at least for the 50 genes included in our panel. Although we have no helpful explanation for this lack of response we can hypothesize that the absence of target mutations may have decreased the therapeutic effectiveness of the multi TKI agents. This phenomenon is for instance well known in the subset of gastro-intestinal stromal tumors that are wild-type for the *KIT* and *PDGFRA* genes and generally do not respond to TKIs such as Imatinib and Sunitinib. [20] Another possible explanation is that other genetic alterations, not included in our mutational panel, may confer resistance to TKIs or mTOR inhibitors.

It is also noteworthy that our data do not seem to confirm the well known role of mutations within the mTOR patway components to predict long term benefit from mTOR inhibitors. [21, 22] This discrepancy could be explained with the little number of cases treated with Temsirolimus as first-line therapy in our series. An interesting aspect of our work is the finding of rare potentially actionable mutations such as FGFR3 and JAK3 for which effective targeted agents are available and currently under investigation in other tumor types

We can therefore conclude that in the absence of established molecular predictors of response to targeted therapies in mRCC an NGS approach may address single patients to basket trials enrolling according to molecular specific alterations regardless of the pathological features.

## References

[1] Siegel RL, Miller KD, Jemal A Cancer statistics, 2016. CA Cancer J Clin.

[2] Basso M, Cassano A, Barone C (2010). A survey of therapy for advanced renal cell carcinoma. Urol Oncol.

[3] Von Roemeling CA, Marlow LA, Radisky DC (2014). Functional genomics identifies novel genes essential for clear cell renal cell carcinoma tumor cell proliferation and migration. Oncotarget.

[4] Gerlinger M, Rowan AJ, Horswell S (2012). Intratumor heterogeneity and branched evolution revealed by multiregion sequencing. N Engl J Med.

[5] Huang Y, Gao S, Wu S (2014). Multilayered molecular profiling supported the monoclonal origin of metastatic renal cell carcinoma. Int J Cancer.

[6] Chen F, Zhang Y, Şenbabaoğlu Y, Ciriello G, Yang L, Reznik E, Shuch B, Micevic G, De Velasco G, Shinbrot E, Noble MS, Lu Y, Covington KR (2016). Multilevel Genomics-Based Taxonomy of Renal Cell Carcinoma. Cell Rep.

[7] Brugarolas J (2014). Molecular genetics of clear-cell renal cell carcinoma. J Clin Oncol.

[8] Powles T Second-line therapy after VEGF targeted therapy in metastatic renal cancer: a law of diminishing returns. Clin Genitourin Cancer.

[9] Cancer Genome Atlas Research Network (2013). Comprehensive molecular characterization of clear cell renal cell carcinoma. Nature.

[10] Linehan WM, Spellman PT, Ricketts CJ, Creighton CJ, Fei SS, Davis C, Wheeler DA, Murray BA, Schmidt L, Vocke CD, Peto M, AA Al Mamun, Shinbrot E, Cancer Genome Atlas Research Network (2016). Comprehensive Molecular Characterization of Papillary Renal-Cell Carcinoma. N Engl J Med.

[11] Dornbusch J, Zacharis A, Meinhardt M, Erdmann K, Wolff I, Froehner M, Wirth MP, Zastrow S, Fuessel S (2013). Analyses of potential predictive markers and survival data for a response to sunitinib in patients with metastatic renal cell carcinoma.PLoS One.

[12] Moch H, Humphrey PA, Ulbright TM, Reuter VE (2016). WHO classification of the tumours of the urinary system and male genital organs. International Agency for Research on Cancer, Lyon.

[13] Durinck S, Stawiski EW, Pavía-Jiménez A, Modrusan Z, Kapur P, Jaiswal BS, Zhang N, Toffessi-Tcheuyap V, Nguyen TT, Pahuja KB, Chen YJ, Saleem S, Chaudhuri S (2015). Spectrum of diverse genomic alterations define non-clear cell renal carcinoma subtypes. Nat Genet.

[14] Noon AP, Vlatković N, Polański R, Maguire M, Shawki H, Parsons K, Boyd MT (2010). p53 and MDM2 in renal cell carcinoma: biomarkers for disease progression and future therapeutic targets?. Cancer.

[15] de Martino M, Gigante M, Cormio L, Prattichizzo C, Cavalcanti E, Gigante M, Ariano V, Netti GS, Montemurno E, Mancini V, Battaglia M, Gesualdo L, Carrieri G, Ranieri E (2013). JAK3 in clear cell renal cell carcinoma: mutational screening and clinical implications. Urol Oncol.

[16] Stoehr CG, Stoehr R, Hartmann A, Hofstaedter F, Junker K, Blaszyk H, Wieland WF, Otto W, Denzinger S, Walter B (2012). Mutational activation of FGFR3: no involvement in the development of renal cell carcinoma. J Cancer Res Clin Oncol.

[17] Porta C, Giglione P, Paglino C Targeted therapy for renal cell carcinoma: focus on 2nd and 3rd line. Expert Opin Pharmacother.

[18] Stoehr CG, Stoehr R, Hartmann A, Hofstaedter F, Junker K, Blaszyk H, Wieland WF, Otto W, Denzinger S, Walter B (2008). Von Hippel-Lindau gene status and response to vascular endothelial growth factor targeted therapy for metastatic clear cell renal cell carcinoma. J Urol.

[19] Song Y, Huang J, Shan L, Zhang HT (2015). Analyses of Potential Predictive Markers and Response to Targeted Therapy in Patients with Advanced Clear-cell Renal Cell Carcinoma. Chin Med J (Engl).

[20] Huss S, Elges S, Trautmann M, Sperveslage J, Hartmann W, Wardelmann E (2015). Classification of KIT/PDGFRA wild-type gastrointestinal stromal tumors: implications for therapy. Expert Rev Anticancer Ther.

[21] Huss S, Elges S, Trautmann M, Sperveslage J, Hartmann W, Wardelmann E Tumor genetic analyses of patients with metastatic renal cell carcinoma and extended benefit from mTOR inhibitor therapy. Clin Cancer Res.

[22] Kwiatkowski DJ, Choueiri TK, Fay AP, Rini BI, Thorner AR, de Velasco G, Tyburczy ME, Hamieh L, Albiges L, Agarwal N, Ho TH, Song J, Pignon JC (2016). Mutations in TSC1, TSC2, and MTOR Are Associated with Response to Rapalogs in Patients with Metastatic Renal Cell Carcinoma. Clin Cancer Res.

